# Biorefinery—inspired, two-step valorization strategy to manage plant-based recalcitrant organic waste, involving solvent extraction, and fermentation with *Bacillus clausii*—a proof of concept study

**DOI:** 10.3389/fmicb.2024.1507918

**Published:** 2025-01-15

**Authors:** Sreejith Meppoyilam, Ajith Madhavan, Chinchu Bose, Jayasree Pooja, Swetha Suresh, Bipin G. Nair, Sanjay Pal

**Affiliations:** School of Biotechnology, Amrita Vishwa Vidyapeetham, Kollam, India

**Keywords:** antimicrobial compounds, biorefinery, fermentation, recalcitrant lignocellulosics, *Allium cepa*, wastewater malodour, *Salmonella enterica*, disinfection

## Abstract

Approximately 40–50% of municipal solid waste is organic and causing biogenic malodor and infections, due to inefficient treatment methods. Biorefinery-based bioremediation and valorization is in vogue against these conventional strategies since it combines unit operations for better efficiency and productivity. Deriving inspiration, the proposed strategy puts together a unique and compatible combination of processes. This novel two-step valorization workflow involves the extraction of small molecules using organic solvents, and fermentation of resulting denatured residues (increased biodegradability or decreased recalcitrance) of reduced microbial load. The extraction step also doubles up as a sterilization event, with different solvents (petroleum ether, chloroform, ethyl methyl ketone and methanol) exhibiting varied efficiency, methanol and ethyl methyl ketone being the most effective. Different recalcitrant plant organic wastes resulting from four plants (*Cocos nucifera*, *Allium cepa*, *Artocarpus hirsutus* and *Swietenia mahagoni*) were used as feedstocks in the preliminary exploratory study using chosen pathogenic bacteria. Onion peel (*Allium cepa*) ethyl methyl ketone extract was chosen for further studies, as it inhibits *Salmonella enterica*, which is associated with infection and malodour (due to biogenic H_2_S) in wastewater. Further, fractionation of the extract yielded quercetin and its glycoside. The onion peel residue, after solvent extraction was fortified with peptone and essential minerals to promote the growth of *Bacillus clausii*. Fortified post-extraction residue supported the growth better than the pre-extraction residue. The residue resultant after solvent extraction was fermented with *Bacillus clausii* and with release of bioactive supernatants. The concentrated supernatant showed significant inhibition of *Salmonella enterica* and *Shigella dysenteriae*. Additionally, all the exudates showed considerable inhibition in H_2_S production, respectively.

## Graphical abstract

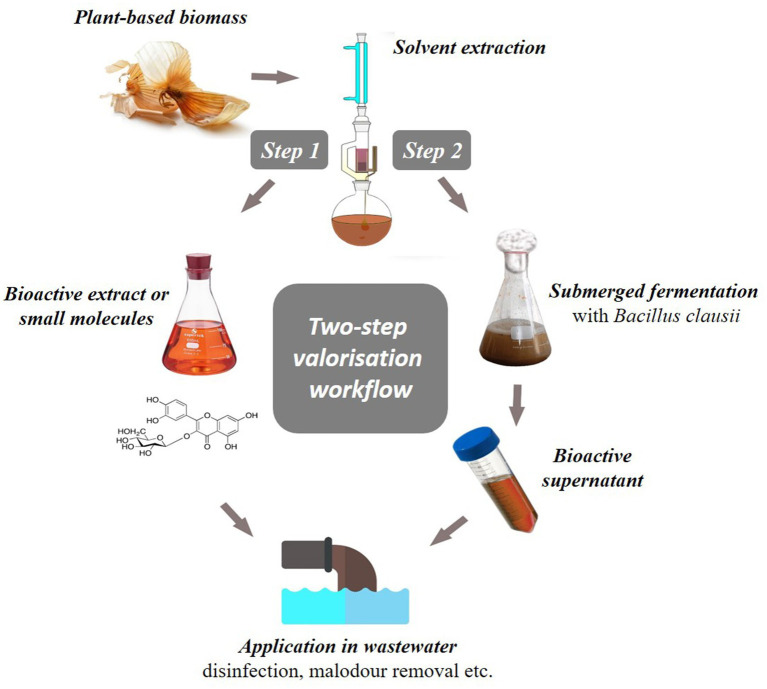

## 1 Introduction

Solid waste management is increasingly becoming challenging. Improper treatment generates hazardous by-products, posing human health hazards and is proving to be deleterious to the environment (Sapuay, 2016). Nearly 2 billion tonnes of municipal solid wastes are generated globally, of which over 30% remains uncollected. Out of the total collected waste, 70% ends up in landfills or dumpsites, close to 19% is recycled, and the remaining 11% is used for energy recovery (Nanda and Berruti, 2021). Organics constitute 40 to 50% of municipal solid waste, mostly biodegradable, and comprise domestic and agricultural waste (Ramachandra et al., 2018). They are predominantly managed through incineration and landfills (Kumar and Agrawal, 2020), with significant environmental costs (Shah et al., 2022).

Composting is touted as a viable alternative, since it ensures valorization, and is environment friendly. However, composting is time-consuming complex microbial process with uncertain quality of resulting fertilizer and hence of low value. It also generates aspirational inconveniences, such as malodor and infection (Sharma et al., 2024; Kumar and Agrawal, 2020). A major reason for the slow process is the recalcitrance of lignocellulosic organic waste (Zoghlami and Paës, 2019; mainly plant based biomass) and the presence of antimicrobial defense metabolites. There are physical and chemical methods (Nait M’Barek et al., 2023) are available for pretreatment of lignocellulosic biomass such as strong acid or alkali or microwave. These treatments reduce the recalcitrance and improve biodegradability. Alternatively, anaerobic digestion is an efficient microbial treatment method for organic waste and nutrient recycling, converting 95% of organic waste into useful products viz., biogases and digestate (Patel et al., 2021). Integration of multiple intermediate steps (biomass pretreatment, fermentation and downstream processing) allows for the generation of value-added products, including biofuels, biodegradable polymers, and enzymes for industrial and medical applications (Patel et al., 2021). Though efficient and well adopted, they suffer from prohibitive cost and difficulties in maintaining optimal operational conditions. Moreover, the system is more applicable for large farms and centralized facilities and improper management of digestate also cause environmental issues (Lin et al., 2018).

On the other hand, wastewater poses different issues concerning sanitation, health, and hygiene, when compared to solid waste. They serve as a conduit for infection and contribute to malnutrition leading to a significant health burden (Subhash et al., 2022). The main cause of infection is improper treatment and discharge of wastewater into the receiving water bodies (Jahne et al., 2017), creating an ideal nutritional ambience for the proliferation of pathogens (Cai and Zhang, 2013). The widely adopted disinfection strategies fall into chemical and physical methods (Al-Gheethi et al., 2018), that suffer from disadvantages such as inefficient removal of pathogens, the release of hazardous disinfection by-products (DBPs), high energy requirements and frequent maintenance (Collivignarelli et al., 2021; Salim et al., 2022a). Another ignored issue associated with wastewater is malodour, which has negative implications on health, hygiene, and sanitation. Malodourants include biogenic hydrogen sulfide, methanethiol, ammonia, dimethyl sulfide, methane, and short-chain fatty acids (Salim et al., 2021; Sarat et al., 2023). Most notable is the lack of effective and affordable methods for malodour mitigation (Cai and Zhang, 2013).

Since, organic solid wastes and wastewater pose unique sets of problems, and their mitigation strategies suffer from myriad insufficiencies, the need of the hour is a biorefinery-like approach that conflates unit operations, and concertedly attempts to address these problems. This concept is gaining traction due to its potential to convert plant biomass into spectrum of high value products (Calvo-Flores and Martin-Martinez, 2022; Kalkanis et al., 2022). For example, 90% of waste from fruit processing industries serves as a feed stock for the extraction of various bioactive molecules such as polyphenols, phytosterols, terpenoids, carotenoids, tocopherols and glucosinolates, alkaloids, Poly Unsaturated Fatty Acids (PUFA) and peptides. These products further find use in therapeutic and healthcare sectors (Ritika et al., 2024). Likewise, onion peel is a major source of bioactive compounds viz., quercetin, quercetin glucoside, isoquercetin, vanillic acid, morin, ferulic acid, kaempferol, isorhamnetin etc. (Kumar et al., 2022). To take a specific example of bioactivity exhibited by the aforementioned bioactive compounds, quercetin is antimicrobial, anti-cancerous and antidiabetic. Alternatively, plant extracts can be used to synthesize engineered nanomaterials (ENMs) such as silver nanoparticles that are effective against MDR (multi drug resistant) plant pathogens. Moreover, their distinctive physicochemical characteristics, potent activity against diverse bacterial, fungal, and viral pathogens, and suitability for use in agricultural environments, position them as promising tools for efficient plant disease management (Kim et al., 2024).

Functionally, biorefinery approach catenates compatible processes to generate valuable products or energy. For example, combining phytochemical extraction with microbial fermentation generates bioactive components that are prospective in terms of economic viability and environmental applications. Compatibility is due to the favorable pre-processing of the feedstock by solvent extraction, rendering it ideal for fermentation through reduction of resident microbial load and substrate modification by the solvent.

In the described study, we propose a novel two-step valorization workflow that can simultaneously manage plant-biomass waste and wastewater disinfection and malodour. The initial step extracts small bioactive molecules from plant-biomass waste using organic solvents, favorably sterilizing (partially) the resultant residues. The study involved lignocellulosic plant biomass viz., *Cocos nucifera*, *Allium cepa*, *Artocarpus hirsutus* and *Swietenia mahagoni*, since they are used at commercial scale in food, and timber industry and generate large volume of recalcitrant organic byproducts, such peels and saw dust. The final step uses the post-extraction this residue (only used *Allium cepa*) after extraction as a feedstock for fermentation by *B. clausii.* The bioactive extracts from the first step and the supernatants from the second hold potential for applications in wastewater remediation. The proposed workflow is also advantageous to its amenability to adopt and process varied plant-biomass waste.

## 2 Materials and methods

### 2.1 Plant materials for the study and solvent extraction

We chose five widely available recalcitrant lignocellulosic plant waste for our study. All plant materials were collected from the vicinity of Vallickavu, Kollam, Kerala, India. Sources of sawdust are coconut (*Cocos nucifera*), wild jack (*Artocarpus hirsutus*) and mahogany (*Swietenia mahagoni*). The endocarp of *Cocos nucifera* and the tunic (peel) of *Allium cepa* were sourced from commercial and domestic kitchens locally (Kollam, Kerala).

All the plant materials (50 g) were dried at 50°C, powdered and sequentially extracted with petroleum ether, chloroform, ethyl methyl ketone (MEK) and methanol (Poulose et al., 2011; Zhang et al., 2018). Subsequently, the solvents were vacuum evaporated at 50°C. Crude extracts were concentrated and dissolved in DMSO (7%) (Vishnu Prasad et al., 2010). The polar extracts (MEK and methanol) were chosen for further studies since they are completely soluble in DMSO and suitable for biological activities. The extracts were coconut shell (*Cocos nucifera*) MEK extract (Coconut shell MEK), coconut shell methanol extract (Coconut shell ME), onion peel (*Allium cepa*) MEK extract (Onion peel MEK), onion peel methanol extract (Onion peel ME), methanol extract of *Cocos nucifera* sawdust (Coconut ME), methanol extract of *Artocarpus hirsutus* sawdust (Wild jack ME) and methanol extract of *Swietenia mahagoni* saw dust (Mahogany ME).

### 2.2 Bacterial strains for study

Spores of poly antibiotic-resistant *Bacillus clausii* (Enterogermina®, Sanofi-Synthelabo) were procured from the medical outlet in Kollam, Kerala, India (Khatri et al., 2019).

Clinical strains of *Salmonella enterica* MW 116733, (Salim et al., 2022b) *Shigella dysenteriae* ATCC 13313, multidrug-resistant *Escherichia coli*, methicillin-resistant *Staphylococcus aureus* and *Klebsiella quasipneumoniae* were procured from Dr. Bhabatosh Das, Associate Professor, Translational Health Science and Technology Institute, Delhi, India (Prasad et al., 2022). *Acinetobacter baumannii* MTCC 1425 and *Pseudomonas aeruginosa* MTCC PA01 were procured from the Microbial Type Culture Collection and Gene Bank, Chandigarh, India (Salim et al., 2022a).

### 2.3 Identification of quercetin and quercetin glycoside from onion peel MEK extract

Literature abounds, indicating the presence of quercetin and its glycoside in onion peels (Choi et al., 2015; Kwak et al., 2017). Hence, the workflow was designed to isolate and identify these molecules from the chosen extract. Onion peel MEK extract was subjected to column chromatography for further purification (Vishnu Prasad et al., 2010). A gradient elution method employed solvents ranging from low to high polarity with varying ratios (Gini and Jeya Jothi, 2018). The solvents used were petroleum ether (PE), ethyl acetate (EA) and methanol (MeOH). The ratios used were 75:25, 50:50, and 25:75 for PE: EA and 98:2, 95:5, 90:10, and 0:100 for EA:MeOH (Kumar et al., 2017). All column fractions were subjected to TLC (Thin layer chromatography) with a solvent system composed of toluene, ethyl acetate and formic acid at a ratio of 50:50:10. The standard used was a mixture of isorhamnetin and quercetin. The nine pooled fractions (A-I) were checked for antimicrobial activity against *S. enterica* and monitored using a resazurin-based viability assay. Among all the fractions, B (EA:PE of 50:50) and H (EA:MeOH of 95:5, 90:10 and 0:100), owing to their respective inhibitory and promotion activity, were further characterized using HPLC (High Performance Liquid Chromatography) and LC–MS (Liquid Chromatography- Mass Spectrometry). HPLC was performed on Shimadzu-SPD-M20A, equipped with DAD (Diode Array detector) with Phenomenex Luna 5 *μ* C_18_ 100A column, with the size of 250 × 4.60 mm dimension. The protocol was performed with acetonitrile solvent systems with 0.3% O-phosphoric acid (Kumar et al., 2017). On the other hand, LC–MS analysis was run on Agilent 1,290 series ultrahigh performance liquid chromatography (UHPLC) coupled to an Agilent ion trap mass spectrometer (6,340 series) with an electrospray interface. LC–MS analysis was run on Agilent 1,290 series ultrahigh performance liquid chromatography (UHPLC) coupled to a mass spectrometer (Agilent 6,540 UHD Accurate Mass Q-TOF) fitted with a dual Agilent Jet Stream with an electrospray ion source (Haripriyan et al., 2018; Nair et al., 2019).

### 2.4 Sterilization efficiency of organic solvents used in the extraction of phytochemicals

Onion peel residue was spiked with 10^5^ spores of *B. stearothermophilus* and dried overnight at 50°C. The dried residue (1 g) was suspended in 10 mL of autoclaved distilled water and spread over nutrient agar plates, this served as the control representing the pre-extraction feed stock. As a test set-up, 1 g of each of the residues was soaked in 10 mL of chosen organic solvents viz., petroleum ether, chloroform, ethyl methyl ketone and methanol for overnight in a shaking incubator. Solvents were completely removed by drying at 50°C in 24 h. The residues were suspended in 10 mL of autoclaved distilled water and spread-plated onto nutrient agar.

### 2.5 Post-extraction residue of onion peel as a media component

Onion peel residue was collected after solvent extraction and air dried. Residue resultant from MEK extraction was used for further studies. The residue (40 g), which served as a carbon source, was fortified with 5 g peptone (HiMedia Laboratories Pvt. Ltd.), 1 g dipotassium hydrogen phosphate (EMPLURA, Merck Life Science Pvt. Ltd.), 0.2 g magnesium sulfate heptahydrate (MERCK Specialities Pvt. Ltd.), 10 g sodium carbonate (Molychem), in order to formulate a media for *B. clausii* (Prasad et al., 2022). The constituted fermentation media was termed post-extracted-onion peel-carbonate fermentation media (POCFM). The submerged fermentation of *B. clausii* was performed with media constituted with pre-extract (ROCFM) and post-extract (POCFM). Bacterial load was monitored at 0, 48 and 72 h.

### 2.6 Fermentation in POCFM with *Bacillus clausii*

The submerged fermentation was carried out at 37°C, 250 rpm for 72 h. The fermentation broth were collected after 48 and 72 h periods for further studies. Following centrifugation (4°C, 7000 RCF, 10 min), the supernatant were concentrated for 5 h at 85–90°C. The concentrated supernatant (Con.OPR48 and Con.OPR72 were collected at 48 and 72 h respectively) were clarified with another round of centrifugation (4°C, 7000 RCF, 10 min).

### 2.7 Antimicrobial activity of plant biomass extracts and concentrated supernatant of *Bacillus clausii*

Resazurin-based viability assay and culture-based assay were performed to check the efficacy of seven plant biomass extracts and nine column fractions on the growth of the targeted pathogenic bacteria. Fluorescence (530/590 nm) were measured at 0, 2, 4, and 6 h and column fractions at a single time point of 4 h (Rampersad, 2012). The culture-based method was also used for all seven extracts, two fractions (B & H), and concentrated supernatants (Con.OPR48 and Con.OPR72) against targeted pathogenic bacteria (as used in resazurin viability assay) at intervals of 0, 6 and 24 h. Sufficient controls were also kept. On the other hand, the antimicrobial activity of concentrated *B. clausii* supernatants (Con.OPR48 and Con.OPR72) were assessed only with culture-based assays since they were colored and interfered with fluorescence readings at 530/590 nm. Bacterial OD (600 nm) was adjusted to 0.3 using a biophotometer (Eppendorf, D30). All experiments were performed in triplicates.

Various concentrations of plant extract, 1.5 to 5.5 mg/mL, were used for resazurine-based viability assay. Additionally, the culture-based assay was performed using concentrations ranging from 4 to 5.5 mg/mL since they showed a significant reduction in the fluorescence assay. MIC_90_ (Minimum inhibitory concentration 90) and MBC (Minimum bactericidal concentration) were determined (Amini Tapouk et al., 2020) from resazurin based viability assay and culture-based assay, respectively.

### 2.8 Estimation of biogenic H_2_S reduction using lead acetate strip assay

A lead acetate test was employed to detect the H_2_S production. The lead acetate-impregnated filter paper turned black due to the formation of lead sulfide when reacted with H_2_S. The blackening of the filter paper is proportional to the concentration of H_2_S (Hine and Mitchell, 2017). The OD of *S. enterica* culture was adjusted to 0.3 at 600 nm. The reaction mixture in 48-well microtiter plates included 420 μL of culture, 138 μL autoclaved distilled water, 42 μL of plant extract (2 mg/mL), and 60 μL of 12 mg/mL L-cysteine. DMSO and culture were maintained as control (Basic et al., 2015). Reaction was carried out at three time points, namely 0, 6 and 24 h. The lead acetate-infused Whatman filter paper was affixed to the individual lids of three 48-well plates, sealed airtight with parafilm, and incubated at 37°C (Salim et al., 2021). The images were captured in a gel doc system (Bio-Rad, United States). ImageJ software was used to measure the H_2_S as the pixel density or Integrated Density (IntDen.) function of the inverted image. The same protocol was followed to check the efficacy of concentrated supernatant on biogenic H_2_S production but with variation in the constitution of the reaction mixture (300 μL bacterial culture, 300 μL concentrated supernatant and 60 μL of 12 mg/mL of L-cysteine). A six-point standard curve was plotted for the assay with a polynomial correlation of Na_2_S concentration versus pixel density (IntDen.) (Supplementary Figure S1). The Na_2_S concentration is the function of the amount of H_2_S released. The range of Na_2_S used for the standard graph was 0.156 to 5 mM.

### 2.9 Statistical evaluation

Graph Pad Prism 8.0.2 was used to carry out statistical analysis. All the experiments were carried out in triplicate. The data were analyzed using one-way ANOVA, two-way ANOVA with Dunn’s multiple comparison tests and Sidak multiple comparison test. The data were also analyzed by Mann Whitney test. All data sets are displayed as a mean with standard deviation.

## 3 Results

### 3.1 Effect of plant extracts against pathogenic bacteria

**Figure 1:**
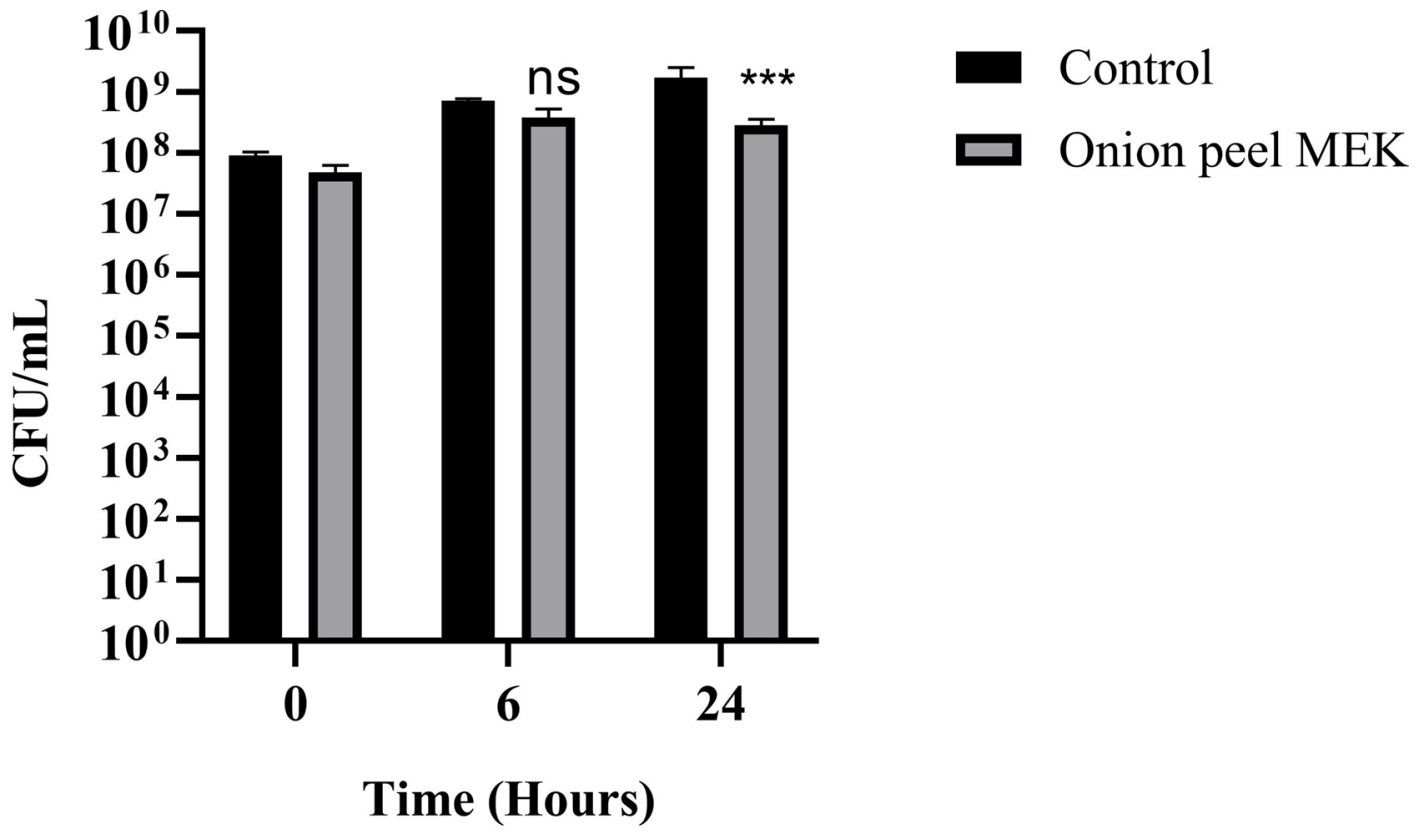
Effect of onion peel MEK extract on *S. enterica*. The effectiveness of onion peel MEK extract against *S. enterica* was measured in CFU/mL, at time intervals 0 h, 6 h, and 24 h. Statistical analysis was performed with the Holm–Sidak’s multiple comparison test (***p < 0.001).

The efficacy of seven plant extracts (coconut shell MEK, coconut shell ME, onion peel MEK, onion peel ME, coconut ME, wild jack ME, mahogany ME) was checked against the targeted pathogenic bacteria viz., *S. enterica, S. dysenteriae, K. quasipneumoniae, A. baumannii, P. aeruginosa,* MDR *E. coli* and MR *S. aureus* using resazurin-based viability assay. Onion peel MEK and coconut shell MEK extracts showed significant inhibition against *S. enterica* and MDR *E. coli,* respectively. The onion peel MEK extract also showed inhibition against MR *S. aureus.* In contrast, wild jack ME extract was effective against MR *S. aureus*, and, in turn, mahogany ME extracts were effective against *P. aeruginosa* (Table 1). Onion peel MEK extract was chosen for further studies owing to its ability to inhibit *S. enterica*, a model organism ideal for establishing the application potential of disinfection and malodour mitigation. A reduction of 83% in CFU/mL was observed after 24 h of incubation on treatment with the extract (Figure 1).

**Table 1:** Screening of antimicrobial activities.

Plant extracts	Bacterial strains						
	EC	AB	SE	SD	KQ	PA	SA
Onion peel MEK	No	No	Yes	No	No	No	Yes
Onion peel ME	No	No	No	No	No	No	No
Coconut shell MEK	Yes	No	No	No	No	No	No
Coconut shell ME	No	No	No	No	No	No	No
Wild jack ME	No	No	No	No	No	No	Yes
Mahagoni ME	No	No	No	No	No	Yes	No
Coconut husk ME	No	No	No	No	No	No	No

Target microbes: EC, multidrug-resistant *Escherichia coli*; AB, *Acinetobacter baumanii*; SE, *Salmonella enterica*; SD, *Shigella dysenteriae*; KQ, *Klebsiella quasipneumoniae*; PA, *Pseudomonas aeruginosa*; SA, methicillin-resistant *Staphylococcus aureus*. Symbols “Yes” and “No” represent inhibition and lack of inhibition, respectively. Statistical analysis was performed by Sidak multiple comparison test. Data with a high significance of *p* < 0.0007 was only considered.

**Figure 2:**
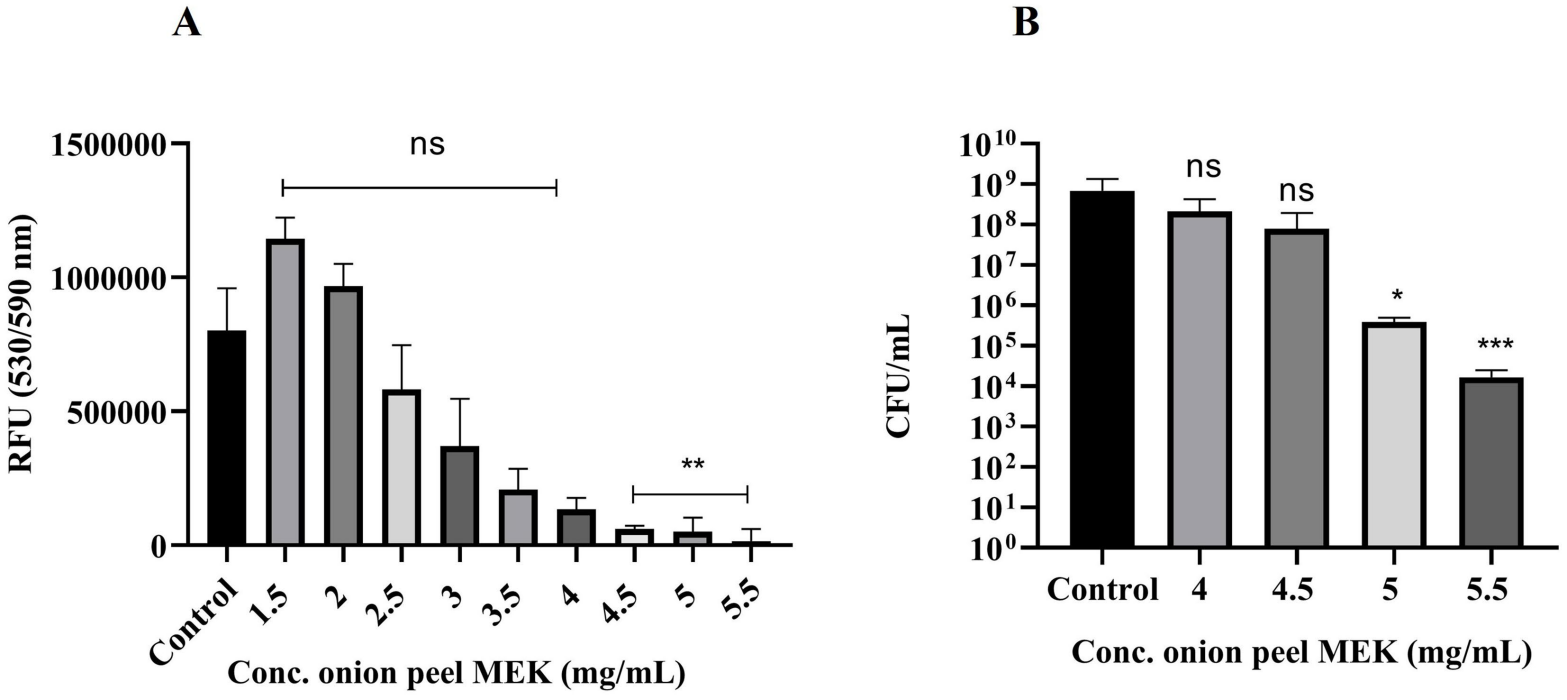
Effect of onion peel MEK extract on *S. enterica*. **(A)** Dose-dependent effect of the plant extract (**p = 0.002) expressed in terms of RFU. **(B)** Different concentrations of onion peel MEK extract expressed in terms of CFU/mL (*p = 0.012, ***p < 0.001). Dunn’s multiple comparison test was used.

Among different concentrations of onion peel MEK extract, ranging from 1.5 to 5.5 mg/mL, the minimum inhibitory concentration required to reduce 90% of the population (MIC_90_) measured in RFU was found to be 4.5 mg/mL (Figure 2A). Furthermore, 4.5, 5 and 5.5 mg/mL concentrations showed 1 log, 3 log and 4 log inhibition in terms of CFU/mL, respectively, (Figure 2B). Hence the MBC of extract was deduced 5 mg/mL, which accounts for the threshold of 99.9% inhibition.

In-between nine column fractions, B showed an 86% reduction in bacterial growth, and on the contrary, fraction H showed promotion (220%), expressed in terms of RFU (Supplementary Figure S2A). The same trend is reflected in terms of CFU/mL, with 1-log reduction (96%) for B and an approximately 12% increase in growth for H (Supplementary Figure S2B).

### 3.2 Estimation of biogenic H_2_S reduction

**Figure 3:**
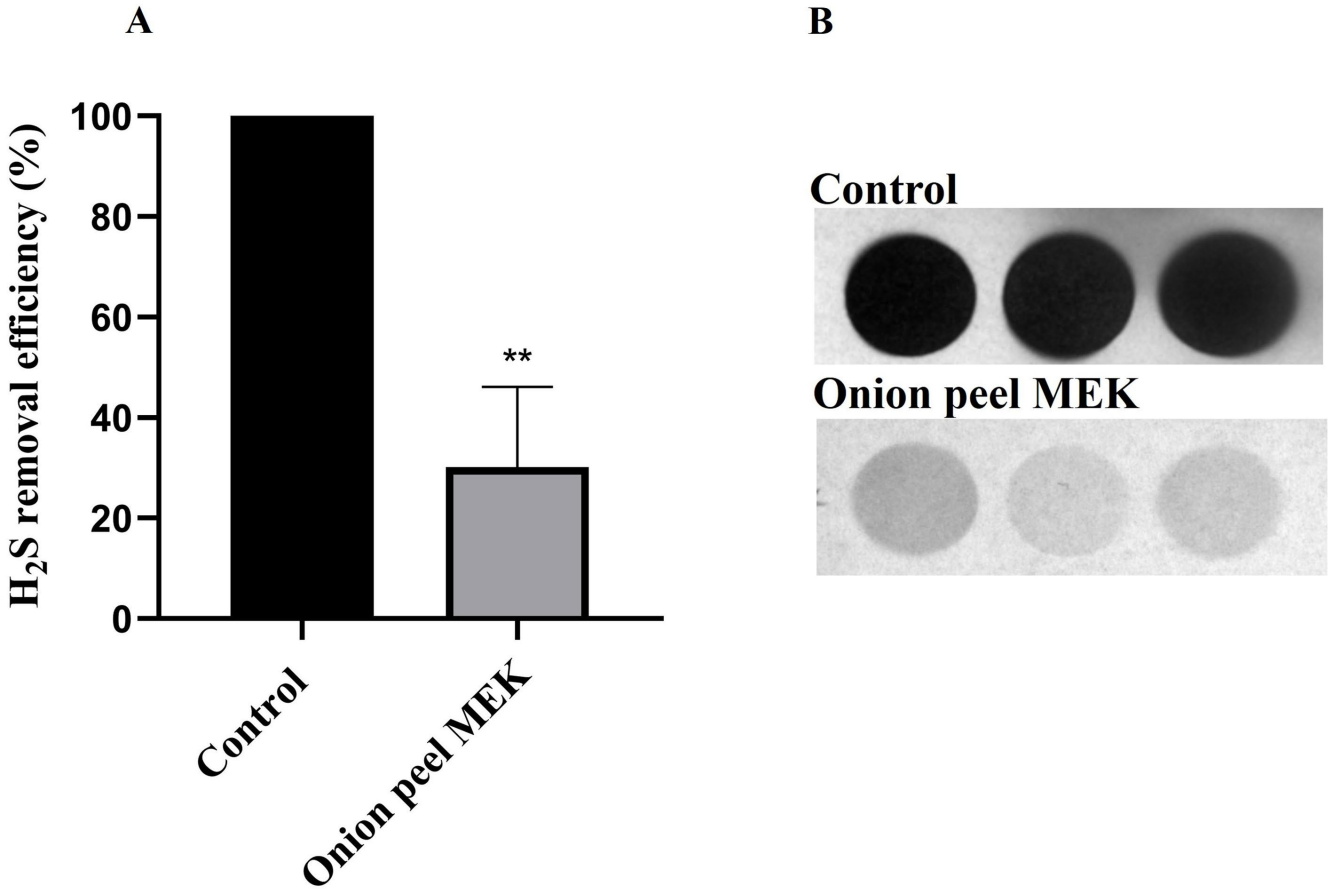
Lead acetate strip-based assay for monitoring the yield of biogenic H_2_S using the Int Den. **(A)** The assay was carried out over 6 h post-treatment time duration and represented in percentage reduction of H_2_S (**p = 0.008). An average of 1.78 mM of H_2_S was considered as 100% in control. **(B)** Image of lead acetate strip showing the difference in the degree of lead sulfide (black color) between control and test post 6 h treatment with onion peel MEK extract. Images were analyzed with ImageJ software. Mann Whitney statistical test was performed to analyses the results.

**Figure 4:**
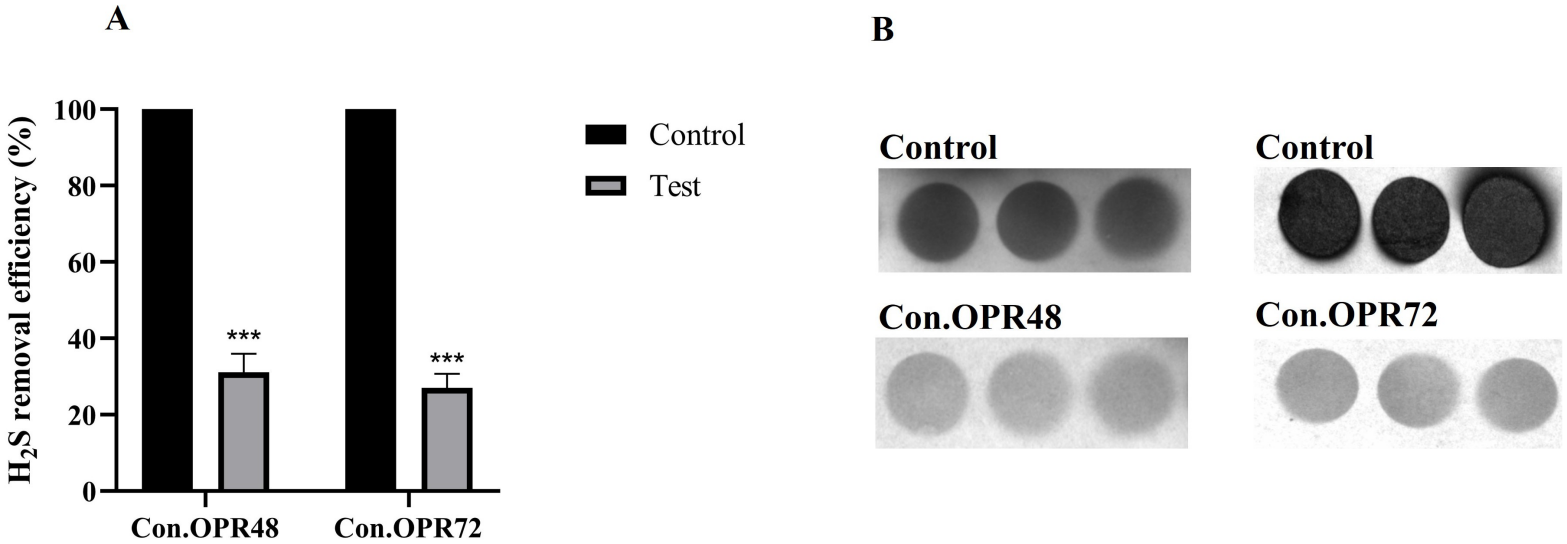
Ability of concentrated supernatants (con. OPR48 and con.OPR72) to reduce biogenic H_2_S. **(A)** Th**e** percentage reduction of biogenic H_2_S by Con.OPR72 and Con.OPR48, post 6 h treatment (***p < 0.001). **(B)** Comparative reduction of H_2_S when compared to control, represented by a reduction in color intensity on lead acetate strip. Images were analyzed with ImageJ software. Sidak’s multiple comparison test was used for analysis.

A lead acetate test was employed to detect the H_2_S production and was expressed in terms of pixel density (IntDen.) Onion peel MEK extract and supernatants (Con.OPR48 and Con.OPR72) showed a significant reduction in biogenic H_2_S post 6 h treatment. Onion peel MEK (2 mg/mL) showed 70% (Figures 3A,B) reduction, and Con.OPR48 and Con.OPR72 showed 69 and 73%, respectively, (Figures 4A,B).

### 3.3 HPLC and LCMS analysis of column fractions B and H

**Figure 5:**
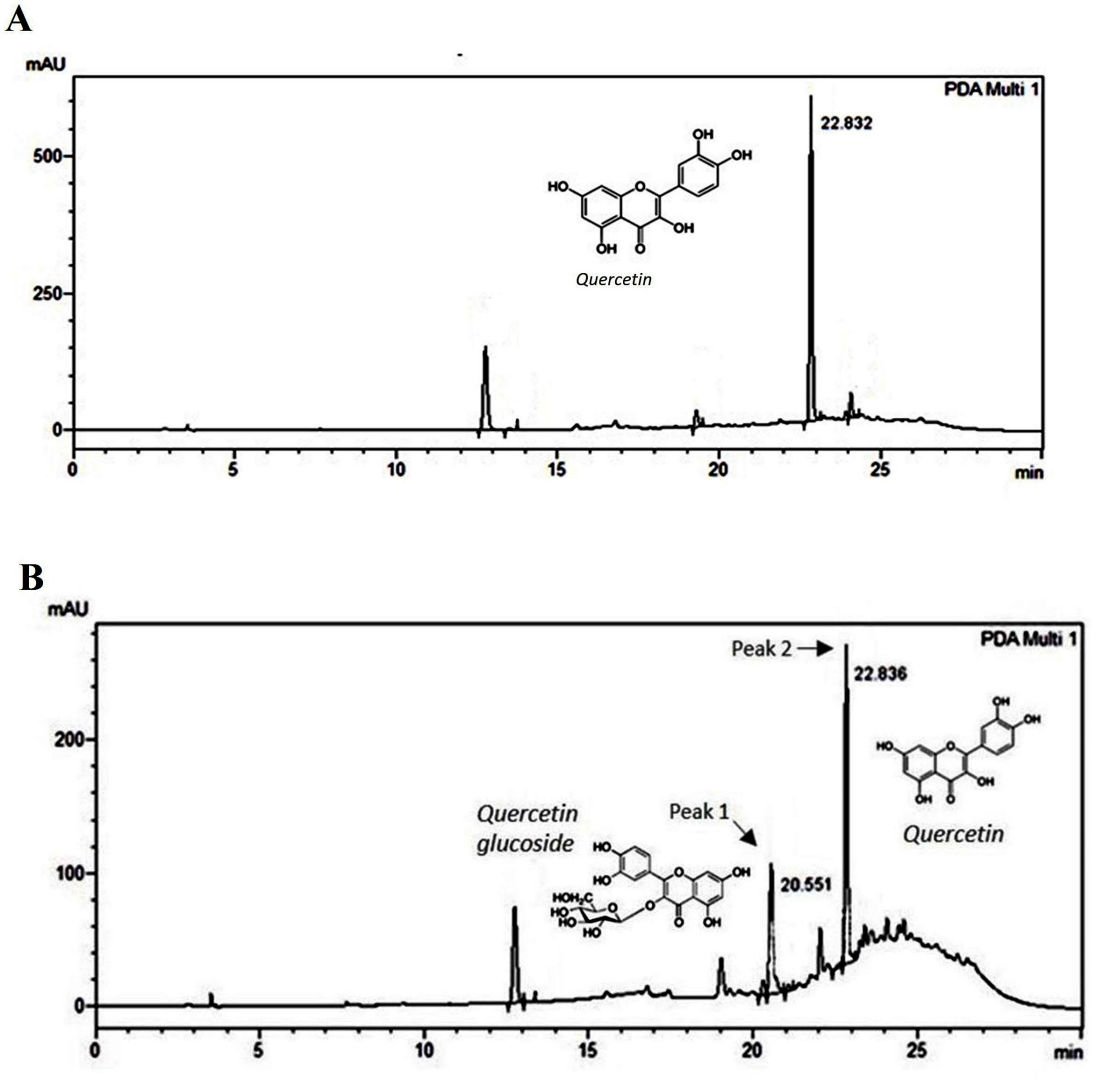
HPLC profile of onion peel MEK column fractions. **(A)** HPLC chromatogram of fraction **B** corresponds to quercetin (UV *λ* max nm: 255, 370). **(B)** HPLC chromatogram of fraction H: Peak 1 (UV λ max nm: 253, 365) and peak 2 (UV λ max nm: 255, 370) representing quercetin glycoside (confirmed as quercetin glucoside by mass spectral analysis) and quercetin.

**Figure 6:**
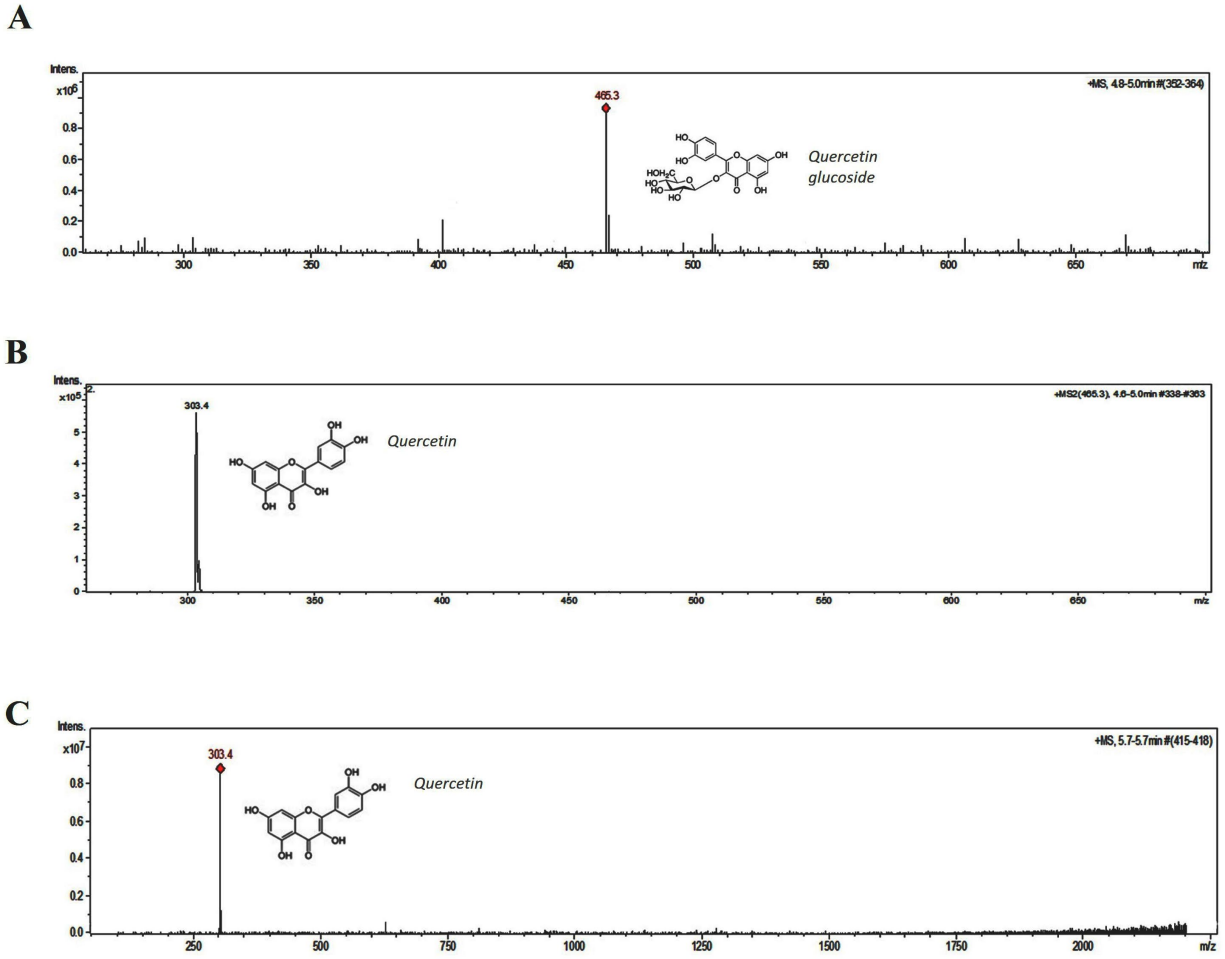
Mass spectral analysis of fraction H. **(A)** Peak 1 with the protonated mass of 465.3 m/z, corresponds to quercetin glucoside. **(B)** MS/MS analysis of 465.3 m/z. **(C)** Peak 2, with the protonated mass of 303.4 m/z, corresponds to quercetin.

The peak (UV *λ* max nm: 255, 370) of fraction B (Figure 5A) corresponds to the peak of quercetin (UV λ max nm: 255, 370) standard (Supplementary Figure S3). On the other hand, apart from the quercetin peak (peak 2), fraction H showed an additional one (peak 1) corresponding to quercetin glycoside (UV λ max nm: 253, 365) further confirmed with MS analysis (Figure 5B). The identity of the peak 1 and 2 of fraction H was corroborated with LC–MS analysis. Protonated *m/z* of 465.3 is the signature mass of quercetin glycoside (Figure 6A). Furthermore, MS/MS analysis of this precursor peak yielded a fragment of 303 *m/z* [M + H], which corresponds to the mass of quercetin (Figure 6B). A mass difference of 161.9 between quercetin and its glycoside indicates that glucose is the sugar moiety. LC- MS analysis of the peak 2 corresponds to the mass of quercetin, 303 *m/z* [M + H] (Figure 6C).

### 3.4 Effect of organic solvents on bacterial growth in onion peel residue

Among the four solvents, MEK and methanol showed a significant reduction in total bacterial load, with the former achieving ~1 log reduction in comparison to ~2 log reduction exhibited by the latter (Supplementary Figure S6).

### 3.5 Post-extraction residue of onion peel as a media component

The residue after organic solvent extraction showed an increased percentage of carbohydrates by ~4% and protein by 0.5% (Supplementary Table S4). The residue thus could potentially serve as a carbon source and hence was included in the formulation of POCFM, which can serve as a fermentation media for *B. clausii*. On component-wise comparison, the residue effectively replaces yeast and starch as carbon sources used in PSCFM (Peptone-Starch-Carbonate-Fermentation-Media) (Supplementary Table S5).

**Figure 7:**
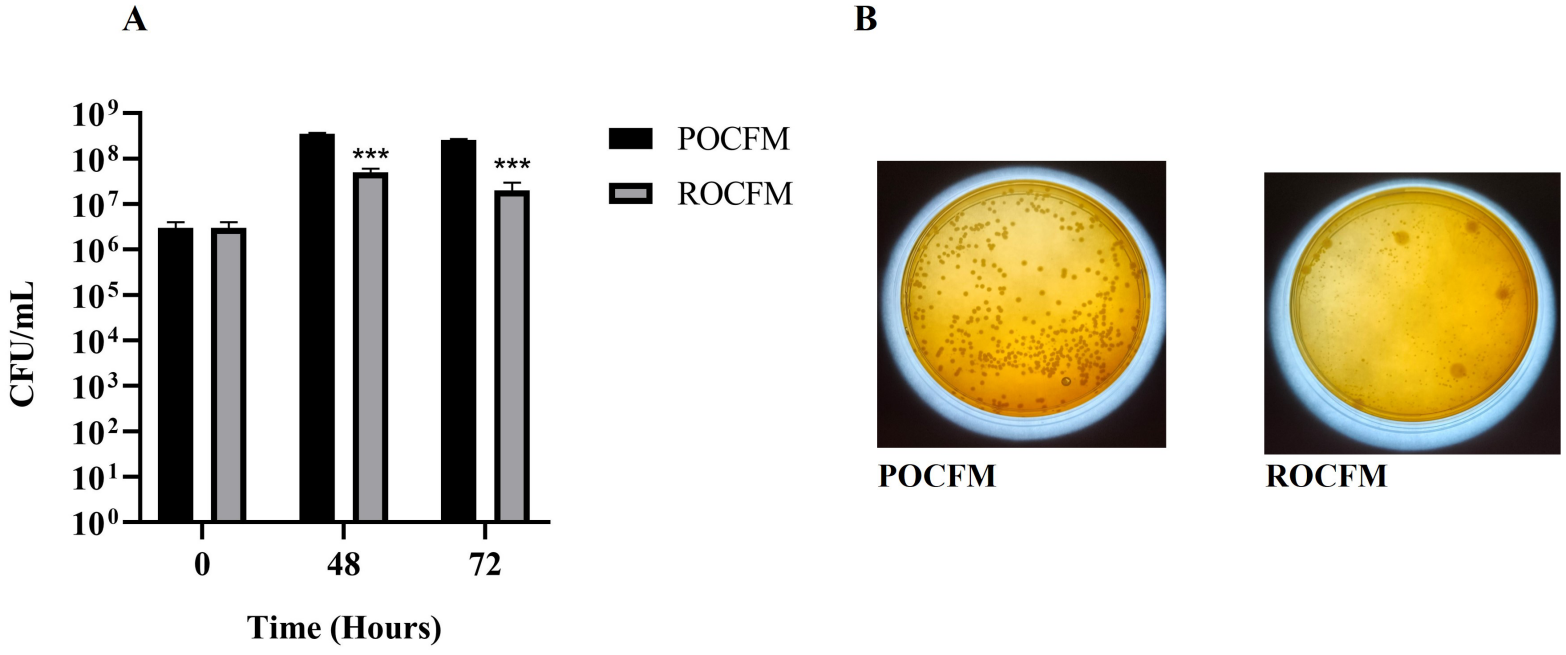
Suitability of pre and post extraction residue of onion peel on the growth of *B. clausii*. **(A)** Growth of *B. clausii* was expressed in terms of CFU/mL in pre extracted onion peel carbonate fermentation media (ROCFM) and post extracted onion peel carbonate fermentation media (POCFM) at 0, 48 and 72 h. **(B)** Images of peptone starch carbonate agar culture plates of ROCFM and POCFM at 72 h. Analysis was done with Sidak’s multiple comparisons test (***p < 0.001).

Fermentation media POCFM supported the growth of *B. clausii* better than the ROCFM. An increase of 1 log and 85%, respectively, were observed with POCFM after 48 and 72 h (Figures 7A,B).

### 3.6 *Bacillus clausii* supernatant on pathogenic bacteria

**Figure 8:**
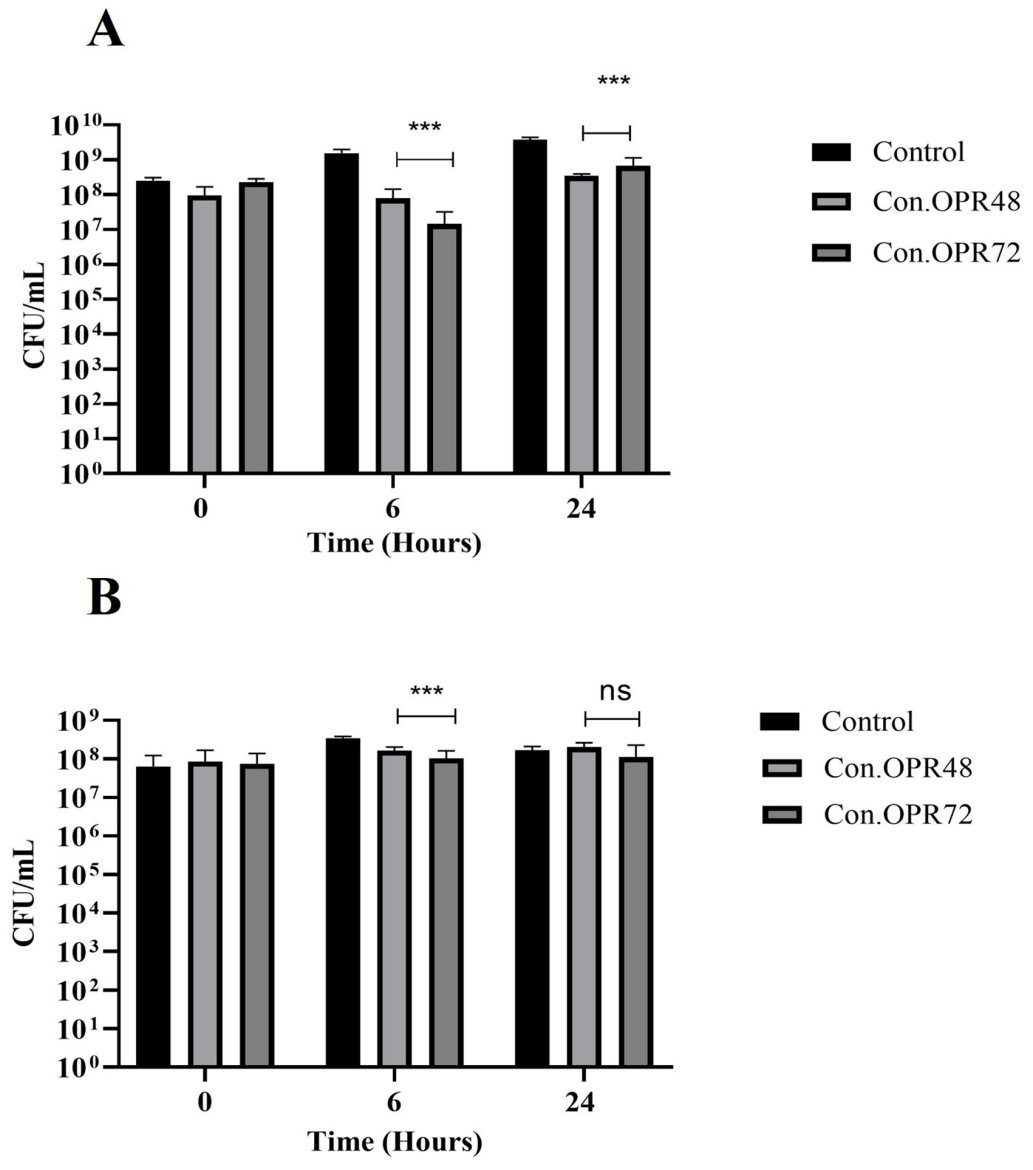
Effect of Con.OPR48 and Con.OPR72 on *S. enterica* and *S. dysenteriae*. Effect on *S. enterica* **(A)** and *S. dysenteriae* **(B)** at 0, 6, and 24 h (***p < 0.001, **p = 0.005). Analysis was done with Sidak’s multiple comparisons test.

The effect of *B. clausii* supernatant was tested against *S. enterica*, *S. dysenteriae, K. quasipneumoniae*, *A. baumanii*, *P. aeruginosa*, MDR *E. coli*, and MR *S. aureus* (Supplementary Figure S7). The supernatants were collected after 48 h and 72 h of incubation and concentrated. Con.OPR72 showed significant inhibition in the growth of *S. enterica* (2 log) and *S. dysenteriae* (70%) after 6 h of incubation. Con.OPR48, in comparison, was less effective with 1 log inhibition in the growth of *S. enterica* and 50% in *S. dysenteriae* (Figures 8A,B).

## 4 Discussion

Solid waste management is in the limelight today, sometimes for the wrong reasons. Improper biowaste treatment may serve as a breeding ground for infection and as a source of malodour (Omang et al., 2021; Shekdar, 2009). The overwhelming volume of biomass, incommensurate with the ability of treatment systems to handle waste, is responsible for improper management and, hence, the associated issues. The proposed study aims to address this issue through a biorefinery-inspired, two-step valorisation process: one involving the isolation of useful small molecules and another enabling efficient treatment and retrieval of bioactive supernatants through submerged fermentation involving *B. clausii*, a GRAS (Generally Recognized as Safe) organism. The solvent extraction process also coincidently, yet favorably sterilizes the residue, rendering it suitable for inoculum addition and controlled fermentation. Moreover, the solvent treatment also improved the availability of carbohydrates, thereby converting the onion peel as a better feedstock for fermentation. Supernatant resultants from fermentation can also be employed for disinfection and malodour mitigation in wastewater (Figure 8).

The proposed strategy is a deviation from conventional physicochemical counterparts in adopting compatible and viable methods which yield useful products and, at the same time, achieve bioremediation objectives, viz. disinfection and malodour removal. The modular nature of the method makes it convenient and amenable for processing different types of agro waste apart from the one used in the study. Moreover, efficient resource recovery associated with this strategy results in better disposal of wastes from plant-based industries. Favorable feedstock modifications rendered by the solvent extraction process through the removal of antimicrobial compounds, such as quercetin (in the case of onion peel) (Nguyen and Bhattacharya, 2022), make them more suitable for fermentation. Additionally, improving unit processes for scale-up will help achieve efficient processing of feedstock, eventually resulting in better bulk reduction.

Infection and malodour are the problems associated with wastewater and have serious implications for health, hygiene, and sanitation. Owing to the ability of plant extracts and bioactive supernatants to reduce pathogens and malodour-causing microbes, makes the strategy suitable for wastewater applications. Restricted selectivity exhibited by the spent media in the inhibition of *S. enterica* and *S. dysenteriae,* when compared to other gram-negative and positive organisms (Supplementary Figure S7), could be advantageous since disinfection and malodour mitigation can be achieved without interfering with the associated bioremediation process. Further corroboration is required to leverage this characteristic effectively.

Onion peel MEK, among different chosen biowaste extracts, showed significant inhibition (MIC_90_ = 4.5 mg/mL) of 1log post 6 h and a 70% reduction in biogenic H_2_S production in post 6 h for 2 mg/mL concentration. The smell reduction is due to the reduction in viable cells of *S. enterica*. The reduced viability could be implicated in the presence of an antimicrobial agent, quercetin (Wang et al., 2018; Zheng et al., 2017). Reports abound on the antimicrobial, antioxidant, antifungal, and antiviral activities of quercetin (Nguyen and Bhattacharya, 2022). An interesting observation on the activity of column fractions, especially H and B, is that they possessed contrasting activity; the former promoted the growth of *S. enterica*, and the latter inhibited it. The presence of quercetin or its glycoside could be attributed to this differential activity (Hervert-Hernández and Goñi, 2011). HPLC and LC–MS data revealed the traces of quercetin in fraction B and its glycoside in fraction H. Inhibitory activity of quercetin and growth promotion characteristics of its glycoside are well documented (Chodak, 2012) and could be responsible for the contrasting activity.

Biorefinery approach demands conflation of compatible unit processes in order to improve its productivity and efficiency (Calvo-Flores and Martin-Martinez, 2022; Chojnacka, 2023). Combining extraction with fermentation provides two advantages viz., sterilization and substrate modification, which are pre-requisite for fermentation. The residue resultant from solvent extraction had sufficient carbon (60.9% carbohydrates) but was limiting in nitrogen (3.85% protein) (Supplementary Table S4). Solvent extraction also marginally improves the nutrients, which, when read along with the ability of the solvents to sterilize the residue partially (Supplementary Figure S6), proves advantageous for submerged fermentation. The residue requires supplementation in order to rectify its nutrient limitations, especially with regards to nitrogen and other macronutrients. These factors play out favorably in the fermentation process improving the growth of *B. clausii* significantly, when compared to the fermentation of raw feedstock (not being subjected to solvent extraction; Figures 7A,B).

Con.OPR72 and Con.OPR48 can also find potential application in wastewater, owing to their antimicrobial activity leading to disinfection and malodour mitigation. The activity could be attributed to hydrolytic enzymes such as proteases present in the supernatant, especially in the stationary phase of bacteria when they undergo metabolic stress because of competition from other bacteria. Interestingly, though, in the study, the protease activity was lost during the concentration of the supernatant. This implies that some other components are responsible for the activity. The supernatant, on the contrary, contains amylase, which is reported to possess biofilm inhibition activity (Lahiri et al., 2021; Solihin et al., 2021) This fact, when read along with the reports on the inhibitory activity of small molecules such as quercetin on amylase, points to favorable feedstock processing through solvent extraction (Pandurangan et al., 2017; Shen et al., 2023; Martinez-Gonzalez et al., 2019). Feedstock devoid of amylase inhibitors will prove conducive for amylase production by *B. clausii*, thereby ensuring antibiofilm activity of the resultant supernatant along with the elusive bactericidal action (Jee et al., 2020).

Inefficient management and disposal of solid waste may lead to environmental and health-related issues. A large proportion of agricultural waste, along with domestic waste, is disposed of improperly, *in situ* or *ex-situ,* such as in landfills, aggravating the issue further. Additionally, improper maintenance and monitoring of landfills generate gasses, viz. methane, carbon dioxide, sulfides and ammonia, contributing to global warming in general and local ecosystem perturbations in specific. Strategies such as this, with trains of modular unit processes with emphasis on valorization, certainly will improve the efficiency of bioremediation, improve economic viability, and leave a minimal environmental footprint.

Finally, like any other, this proof-of-concept study, invokes questions on its amenability for translation. The proposed strategy leans heavily on the concept of biorefinery and adopts features that are successfully translated. Large scale solvent extraction with different feedstocks, is carried out at industrial scale, with efficient solvent recovery and reuse (Isci and Kaltschmitt, 2022; Patra et al., 2022). Similarly, fermentation is also well established and adopted rampantly for large-scale applications. For example, 20 million liters of ethanol is generated from about 100 thousand tonnes of wheat straw, in a facility at Khuzestan, a wheat growing province of Iran (Hasanly et al., 2018). In United States, more than 10 gigalitres of bioethanol is generated from 84 million tonnes of corn stover (Karimi Alavijeh and Karimi, 2019). Catenation of these processes presents advantages in terms of enhanced valorisation and improves the associated processes and thereby making them synergistic.

## 5 Conclusion

Potential commercial viability and customizability for varied applications, makes the described strategy promising for scale up and eventual deployment. This biorefinery-inspired approach involves choosing unit processes that complements each other. Solvent extraction of onion peel for example, renders them partially sterile and amenable for the next step, which is fermentation. Moreover, retrieval of quercetin and its glycosides processes the feedstock by removing these antimicrobial compounds thereby preparing the substrate for efficient fermentation. The study establishes the proof of concept through the isolation of quercetin and its glycoside from onion peel waste in the first step, followed by the generation of a bioactive supernatant through fermentation with *B. clausii*. The onion peel residue after solvent extraction was rendered partially sterile and more suitable for the next step. The solvent treatment also improved the availability of carbohydrates, thereby promoting the fermentation process. In concordance with that, post extracted onion peel residue fermentation media (POCFM) promoted *B. clausii* better than its counterpart, raw onion peel fermentation media (ROCFM). An increase of 1 log and 85% were observed after 48 and 72 h respectively, when compared to ROCFM. Both the MEK extract, and bio active supernatants hold the potential for disinfection and malodour reduction in the wastewater. Maximum of 4 log inhibition was achieved with 5.5 mg/mL of onion peel MEK extract against *S. enterica*. There was an associated significant reduction of biogenic H_2_S production by 70% (at sub MIC_90_ concentration of 2 mg/mL). Additionally, concentrated bioactive supernatants, Con.OPR48 and Con.OPR72 showed inhibition in the growth of *S. enterica* and *S. dysenteriae* after 6 h of incubation, with the former being less effective than the latter. Like the extracts, supernatants also showed effective reduction in the emanation of biogenic H_2_S. In conclusion, the modular nature of the proposed strategy will make it amenable for adoption in the management of various other plant-based organic wastes, other than the onion peel used in the study.

## Data Availability

The original contributions presented in the study are included in the article/Supplementary material, further inquiries can be directed to the corresponding authors.
