# Supplementary material for: Biorefinery—inspired, two-step valorization strategy to manage plant-based recalcitrant organic waste, involving solvent extraction, and fermentation with Bacillus clausii—a proof of concept study
Source: Front Microbiol. 2025 Jan 15;15:1507918. doi: 10.3389/fmicb.2024.1507918 (PMC11781296; doi:10.3389/fmicb.2024.1507918)
Supplement: Supplementary file 1 [file Data_Sheet_1.docx]

Supplementary Material


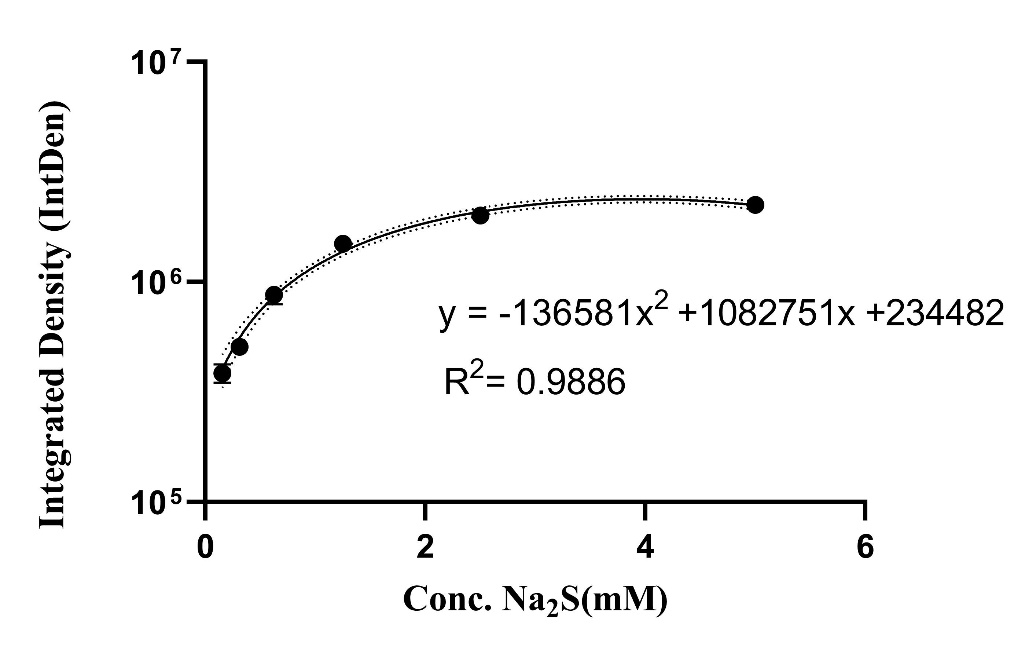


**Supplementary Figure S1** Na_2_S standard graph for monitoring H_2_S concentrations, which was expressed in terms of IntDen.


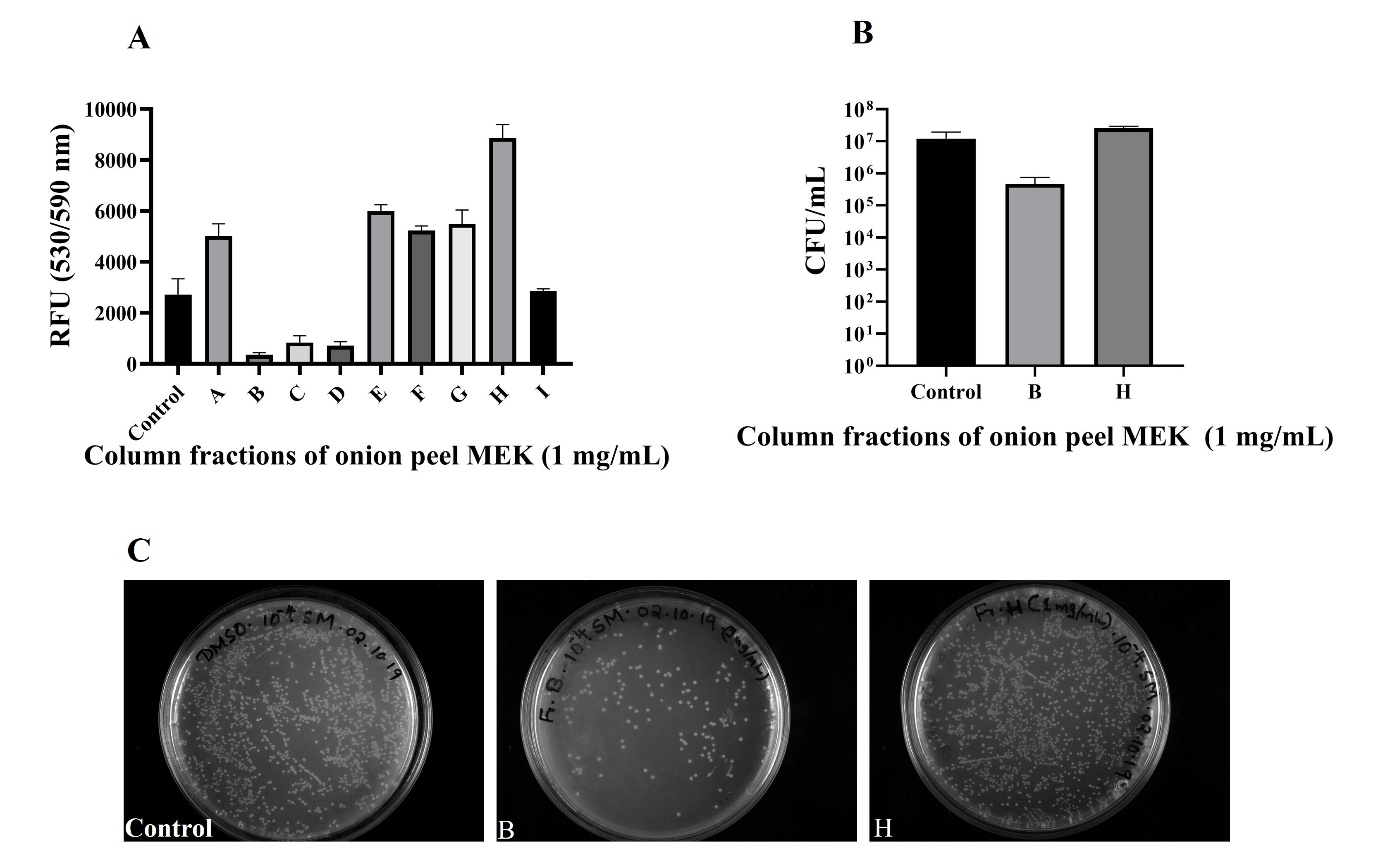


**Supplementary Figure S2** Effect of column fractions of onion peel MEK extract on *S. enterica***.** **(A)** Efficacy of the column fractions (A-I) expressed in RFU. **(B)** Effect of B and H (since they showed maximum inhibition and promotion, respectively) fractions expressed in CFU/mL. **(C)** Representative images of nutrient agar plates showed the effect of B and H fractions with control.


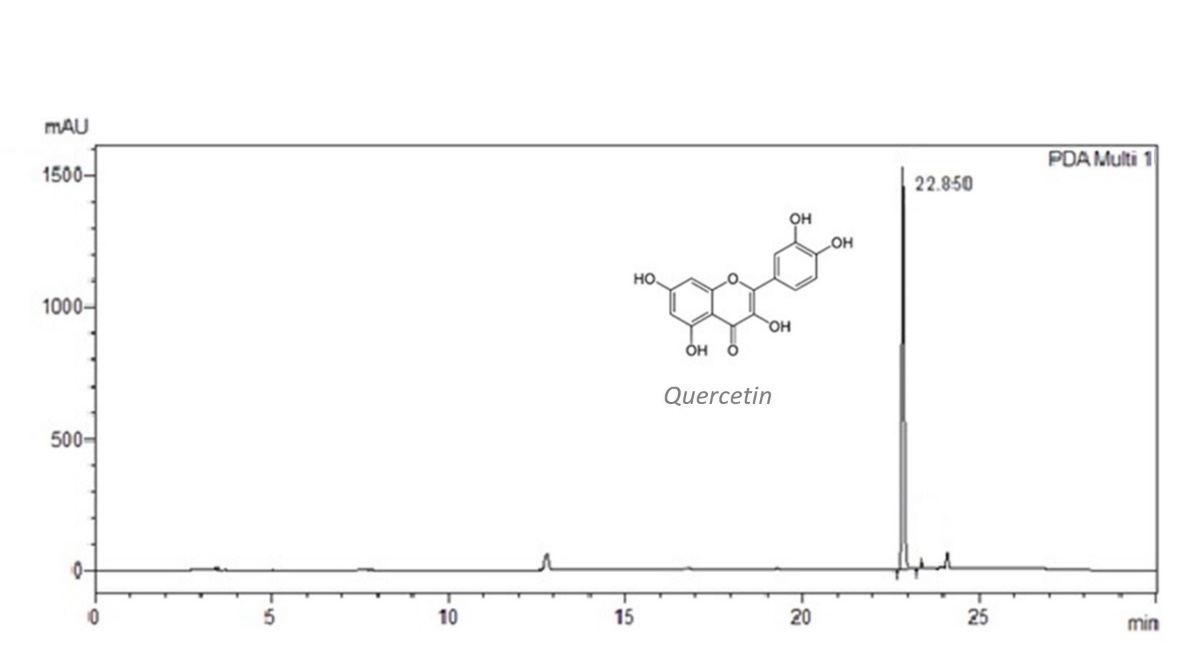


**Supplementary Figure S3** HPLC profile of quercetin standard. The peak was observed at UV λ max nm: 255, 370.

**A**


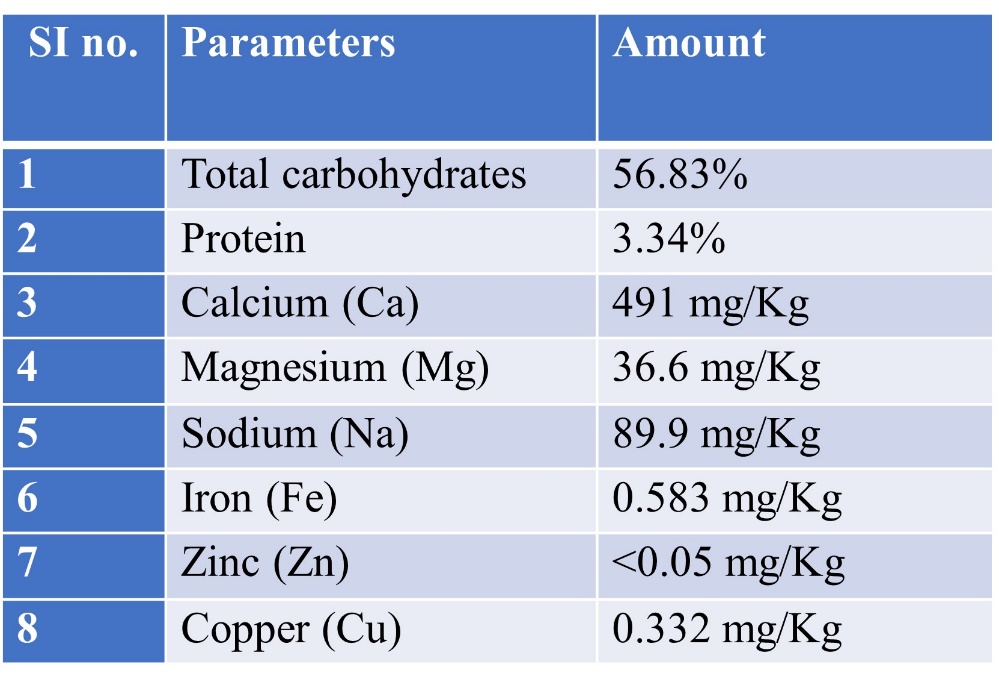


**B**


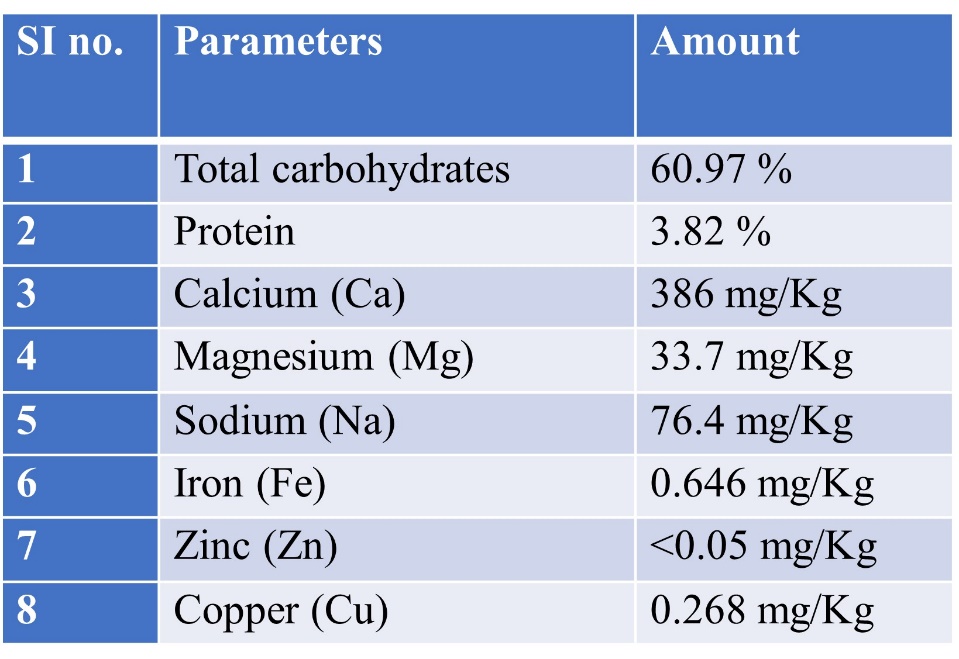


**Supplementary Table S4** pre-and post-compositional analysis of onion peel (*Allium cepa*) employed for solvent extraction. **(A)** Analysis before solvent extraction. (**B)** Analysis after solvent extraction.


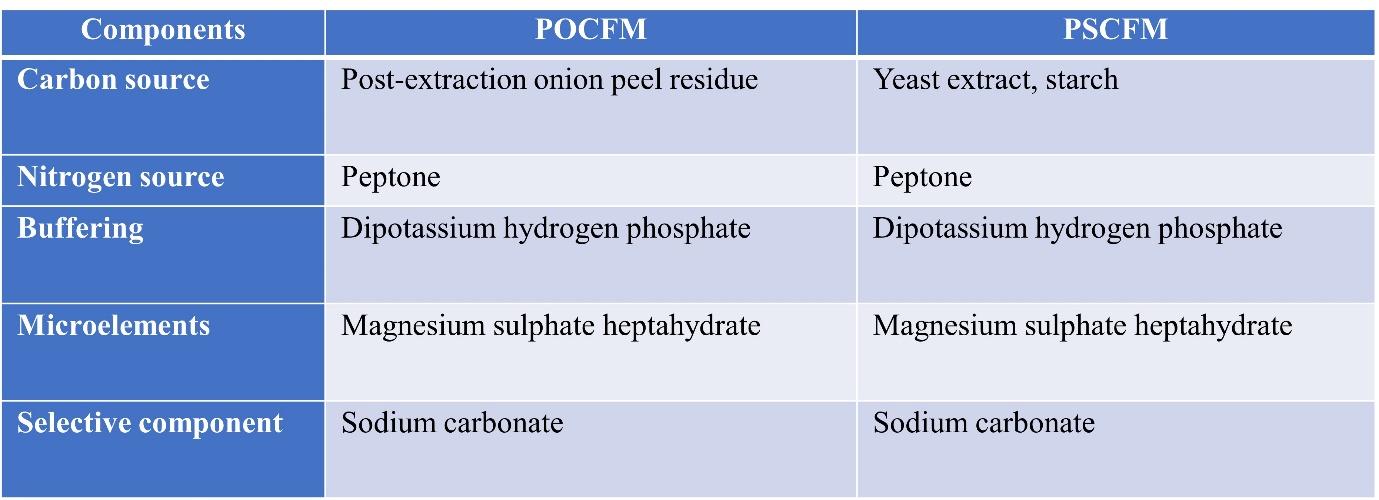


**Supplementary Table S5** Comparison of Post-extracted Onion peel-Carbonate-Fermentation Media (POCFM) with Peptone-Starch-Carbonate-Fermentation Media (PSCFM).


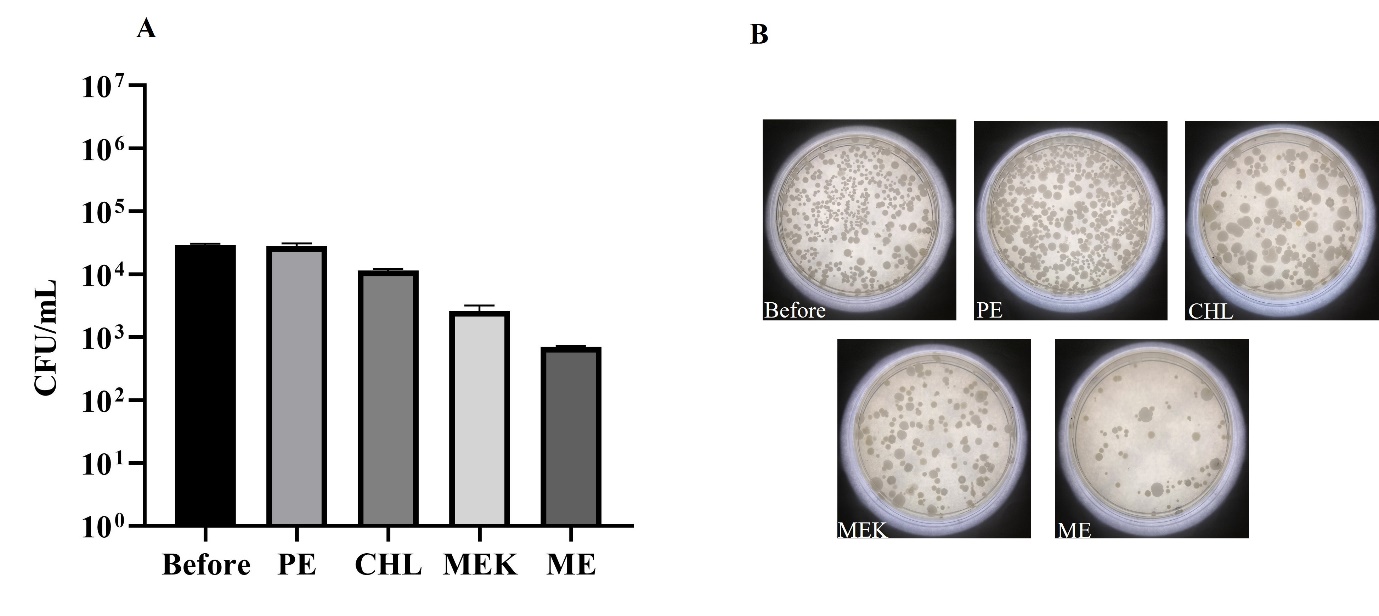


**Supplementary Figure S6** Effect of organic solvents on bacterial growth in onion peel residue. (**A**) Total bacterial count in onion peel spiked (*B. stearothermophilus*) residue before and after solvent extraction (PE: Petroleum ether, CHL: Chloroform, MEK: Ethyl methyl ketone and ME: Methanol) in terms of CFU. (**B)** Representative images of nutrient agar plates.


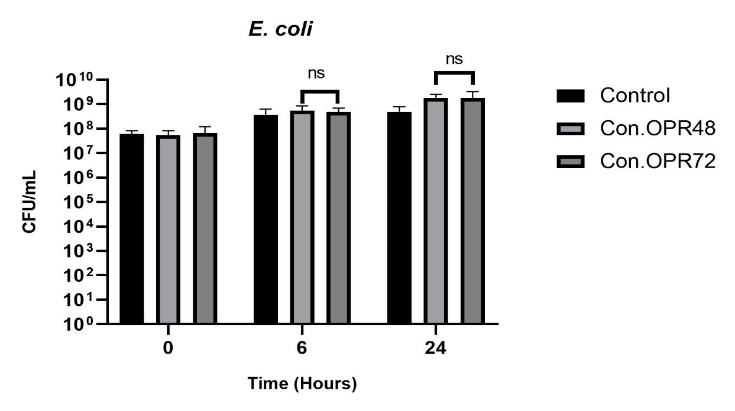


**A**


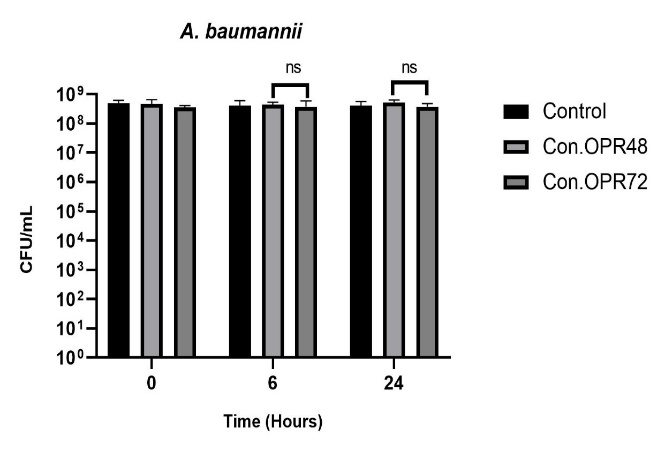


**B**


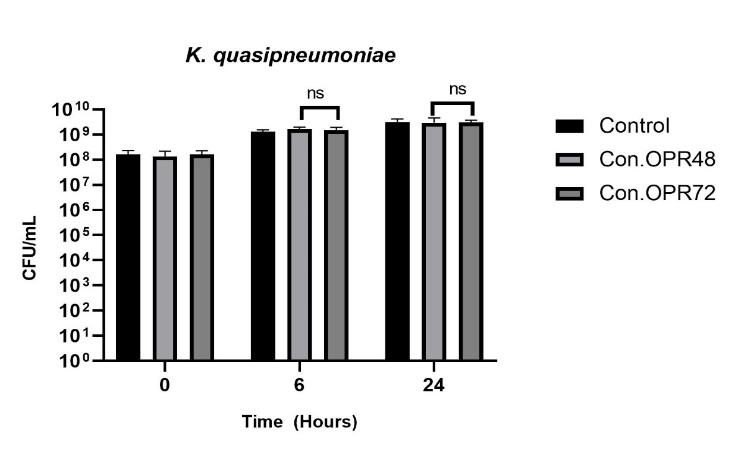


**C**


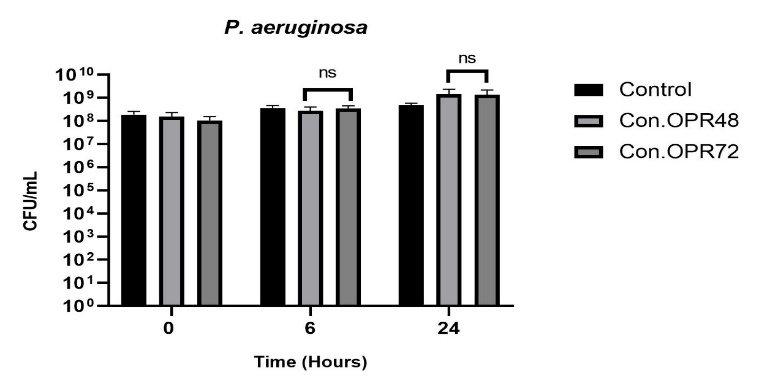


**D**


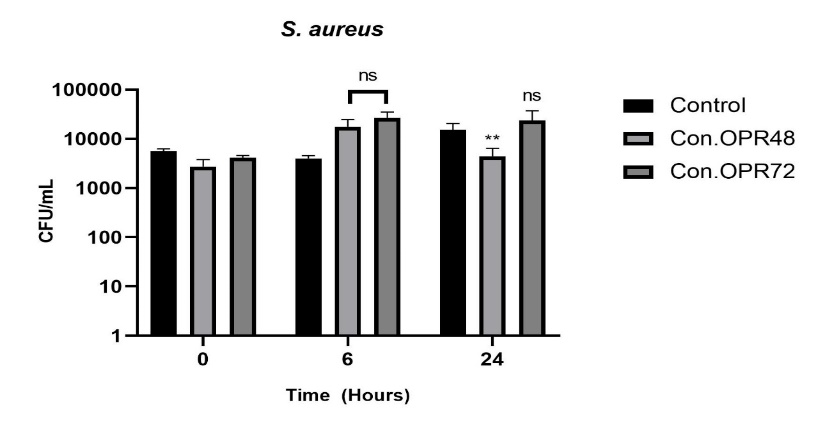


**E**

**Supplementary Figure S7** Effect of Con. OPR48 and Con. OPR72 against selected bacteria. **(A)** *E.coli***. (B)** *A. baumannii***. (C)** *K. quasipneumoniae*. **(D)** *P.aeruginosa.* **(E)** *S. aureus.* Statistical analysis was performed using Sidak’s multiple comparison test (**p = 0.006).


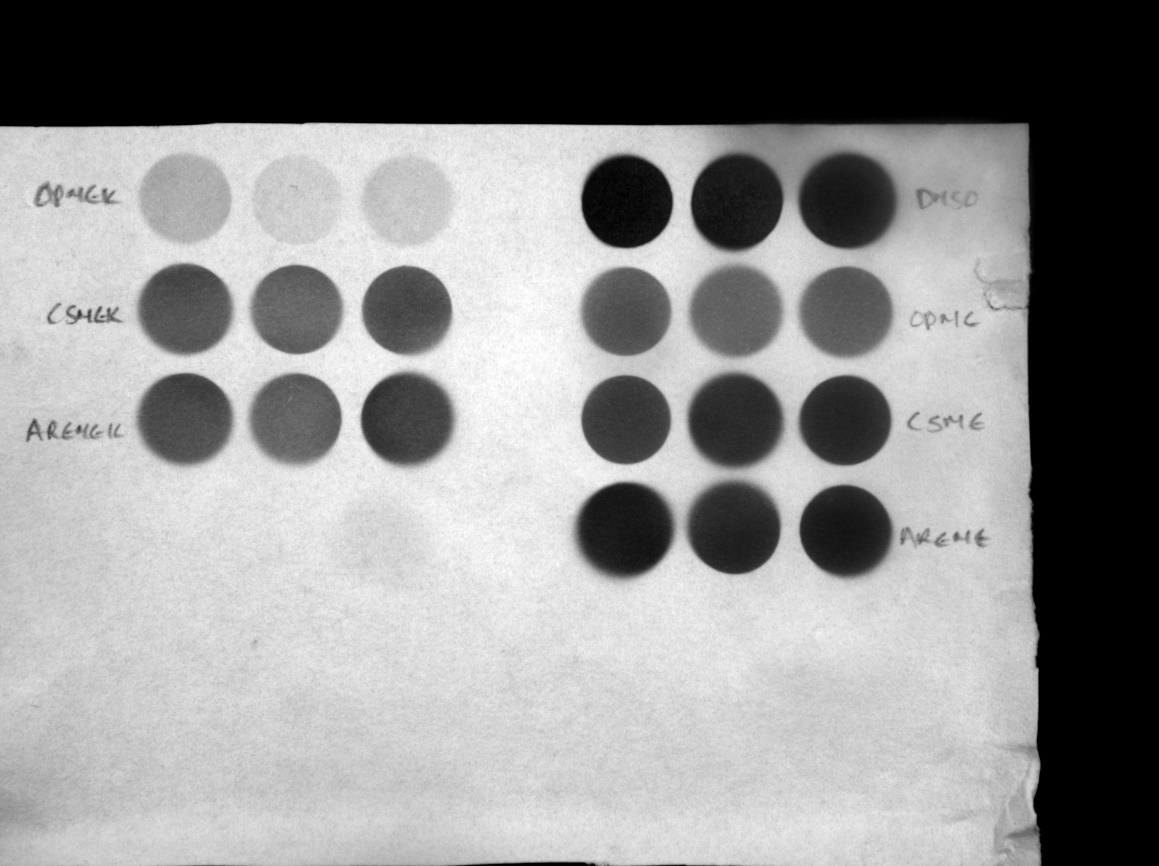


**Supplementary Figure S8** Raw data of onion peel MEK Vs biogenic H_2_S production.


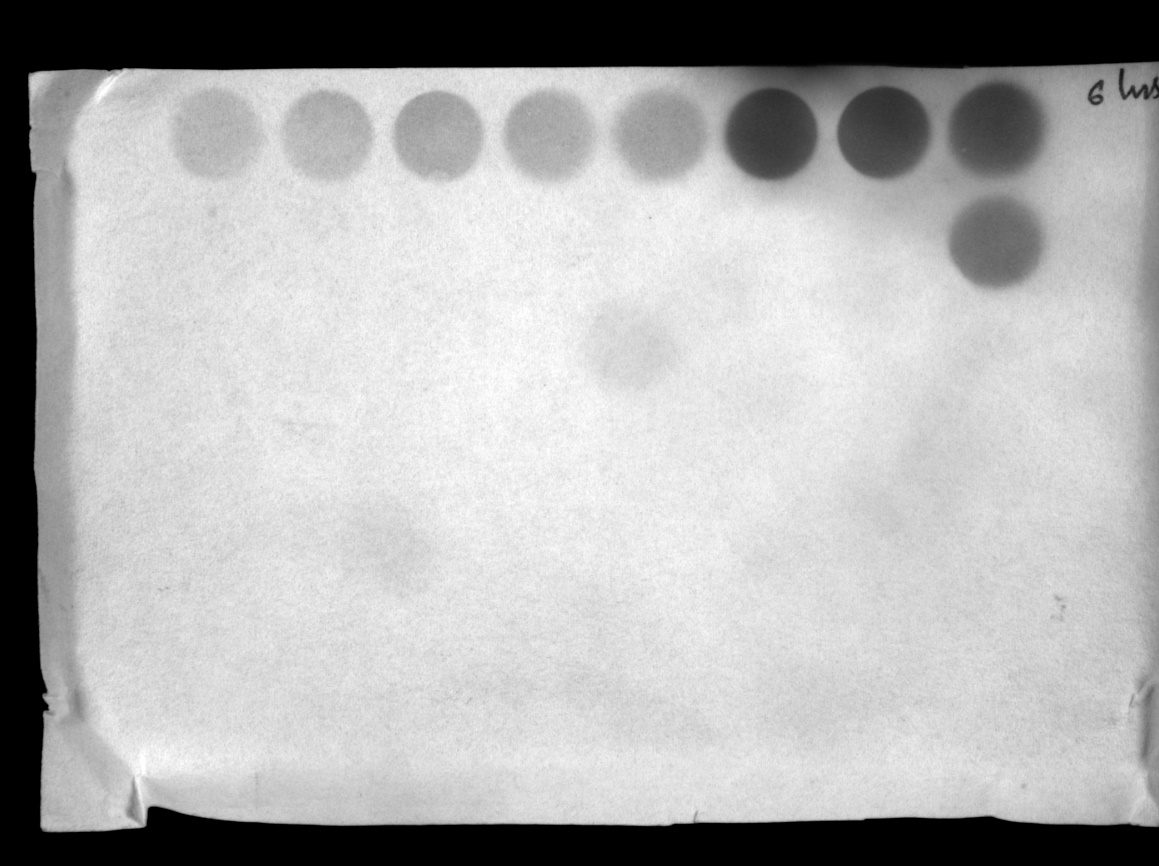


**Supplementary Figure S9** Raw data of Con.OPR48 Vs biogenic H_2_S production.


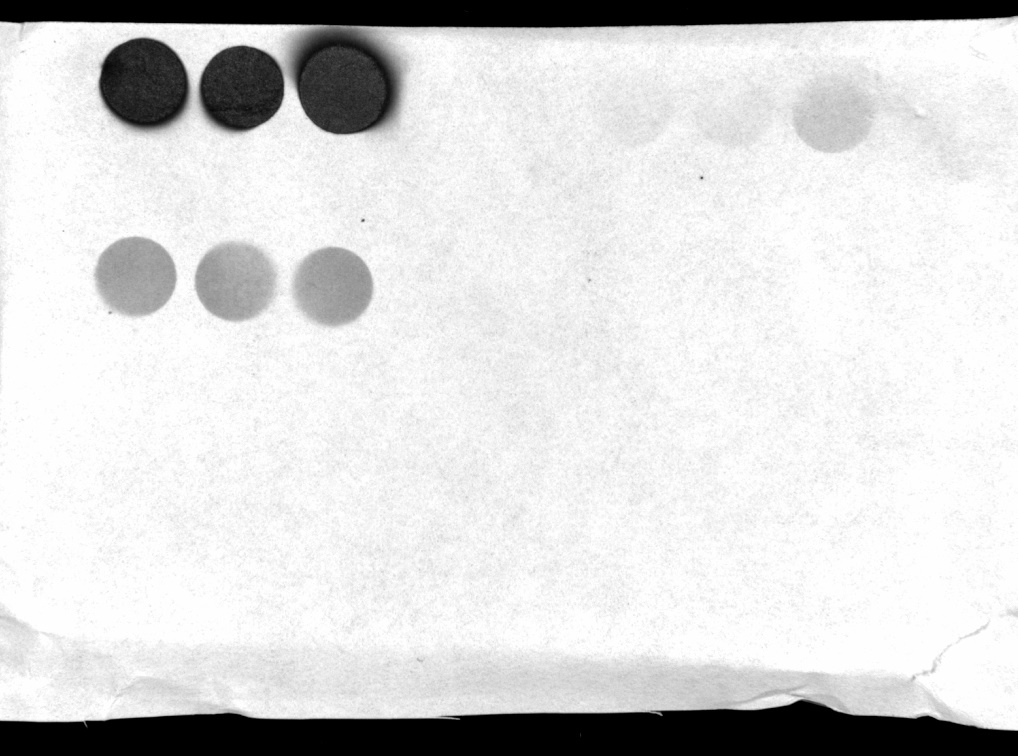


**Supplementary Figure S10** Raw data of Con.OPR72 Vs biogenic H_2_S production.


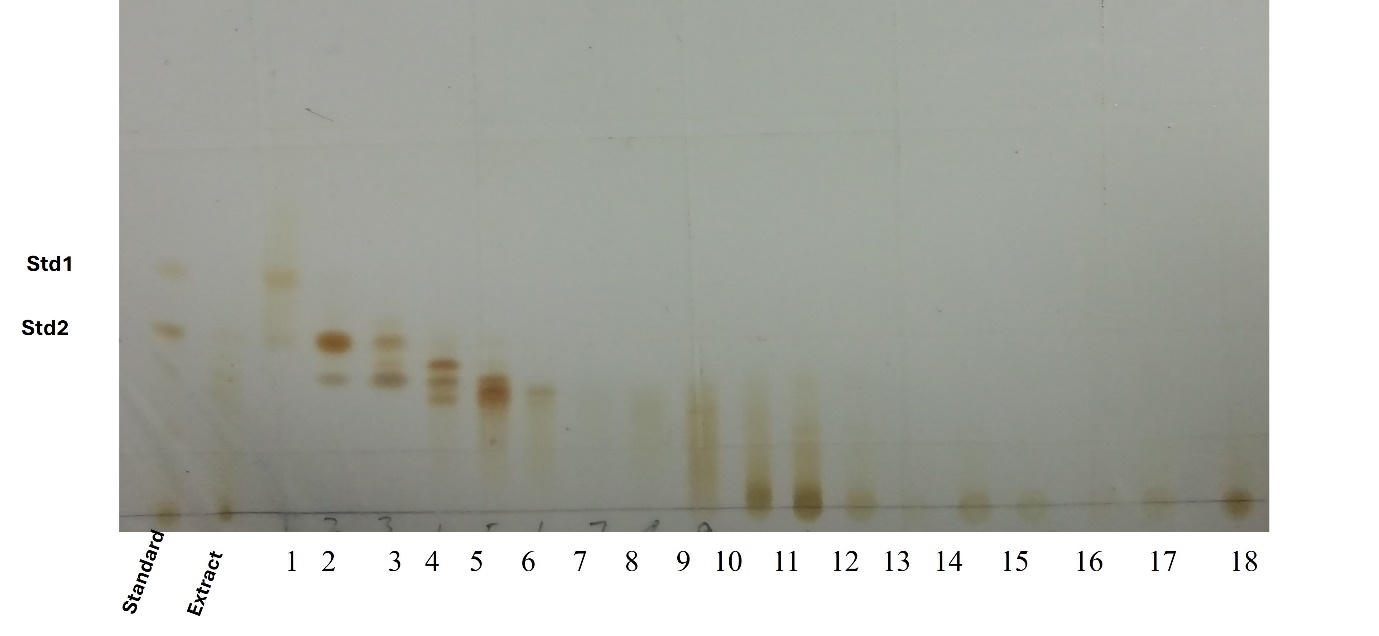


**Supplementary Figure S11** Column fractionation of onion peel( *Allium cepa)* MEK. Std1 is isorhamnetin and Std2 is quercetin.
